# The effect of intranasal oxytocin application and mindfulness-based group therapy for patients with schizophrenia spectrum disorders – A study protocol

**DOI:** 10.1192/j.eurpsy.2022.1969

**Published:** 2022-09-01

**Authors:** M. Zierhut, V. Von Eisenhart-Rothe, S. Graesser, N. Hartter, J. Wohlthan, I. Hahne, N. Bergmann, T.M.T. Ta, M. Bajbouj, E. Hahn, K. Boege

**Affiliations:** 1 Charité - Univerversitätsmedizin Berlin, Campus Benjamin Franklin, Department For Psychiatry And Psychotherapie, Berlin, Germany; 2 Berlin Institute of Health at Charité – Universitätsmedizin Berlin, Bih Academy, Clinician Scientist Program, Berlin, Germany

**Keywords:** Mindfulness, schizophrénia, Oxytocin, negative symptoms

## Abstract

**Introduction:**

Research indicates improvements in negative symptoms and empathy for schizophrenia spectrum disorders (SSD) after mindfulness-based interventions (MBI). Current treatment approaches for SSD remain limited regarding their effectiveness on negative symptoms and sociocognitive deficits. After oxytocin (OXT) administration, especially in a positive social context, an increase in empathy could be shown. The effect of mindfulness in combination with OXT has not yet been examined.

**Objectives:**

This study investigates the additional effect of OXT administration combined with MBI on empathy and negative symptoms in patients with SSD.

**Methods:**

An experimental, randomised, triple-blinded, placebo-controlled study is proposed. Based on power calculations, 140 participants with SSD will be recruited at Charité – Universitätsmedizin Berlin. A dose of intranasal oxytocin with 24 I.U. or placebo will be administered 45 minutes before each session. Following each administration, a total of four MBI interventions will take place for two weeks. Empathy as primary outcome will be measured using validated psychometric questionnaires. Outcomes, including negative symptoms and OXT plasma levels, will be measured at baseline and post-intervention. A 2x2 mixed-model ANCOVA design with time as within- and group as between-subject factor will be calculated to assess empathy and negative symptom changes.

**Results:**

The study hypothesises that applying intranasal oxytocin in combination with MBI will increase empathy and reduce negative symptoms in patients with SSD.

**Conclusions:**

Findings could provide insight into enhancing therapies like MBI by utilising OXT as a possible supplementary treatment option. Findings could therefore pave the way for a personalised psychiatric medicine treatment for individuals with SSD.

**Disclosure:**

No significant relationships.

